# Proteomic Comparison of Malignant Human Germ Cell Tumor Cell Lines

**DOI:** 10.1155/2019/8298524

**Published:** 2019-09-03

**Authors:** Felix Bremmer, Hanibal Bohnenberger, Stefan Küffer, Thomas Oellerich, Hubert Serve, Henning Urlaub, Arne Strauss, Yasmine Maatoug, Carl Ludwig Behnes, Christoph Oing, Heinz Joachim Radzun, Philipp Ströbel, Stefan Balabanov, Friedemann Honecker

**Affiliations:** ^1^Institute of Pathology, University Medical Center, Robert-Koch-Str. 40, 37075 Göttingen, Germany; ^2^Department of Medicine II, Hematology/Oncology, Goethe University, Theodor-Stern-Kai 7, 60590 Frankfurt, Germany; ^3^German Cancer Research Center and German Cancer Consortium, 69120 Heidelberg, Germany; ^4^Bioanalytical Mass Spectrometry Group, Max Planck Institute for Biophysical Chemistry, Am Fassberg 11, 37077 Göttingen, Germany; ^5^Bioanalytics, University Medical Center, Robert-Koch-Str. 40, 37075 Göttingen, Germany; ^6^Department of Urology, University Medical Center, Robert-Koch-Str. 40, 37075 Göttingen, Germany; ^7^Department of Oncology, Hematology and Bone Marrow Transplantation with Section of Pneumology, University Medical Center Hamburg-Eppendorf, Martinistraße 52, 20246 Hamburg, Germany; ^8^Division of Hematology, University Hospital Zurich, Rämistrasse 100, 8091 Zürich, Switzerland; ^9^Tumour and Breast Center ZeTuP St. Gallen, Rorschacher Strasse 150, 9006 St. Gallen, Switzerland

## Abstract

Malignant germ cell tumors (GCT) are the most common malignant tumors in young men between 18 and 40 years. The correct identification of histological subtypes, in difficult cases supported by immunohistochemistry, is essential for therapeutic management. Furthermore, biomarkers may help to understand pathophysiological processes in these tumor types. Two GCT cell lines, TCam-2 with seminoma-like characteristics, and NTERA-2, an embryonal carcinoma-like cell line, were compared by a quantitative proteomic approach using high-resolution mass spectrometry (MS) in combination with stable isotope labelling by amino acid in cell culture (SILAC). We were able to identify 4856 proteins and quantify the expression of 3936. 347 were significantly differentially expressed between the two cell lines. For further validation, CD81, CBX-3, PHF6, and ENSA were analyzed by western blot analysis. The results confirmed the MS results. Immunohistochemical analysis on 59 formalin-fixed and paraffin-embedded (FFPE) normal and GCT tissue samples (normal testis, GCNIS, seminomas, and embryonal carcinomas) of these proteins demonstrated the ability to distinguish different GCT subtypes, especially seminomas and embryonal carcinomas. In addition, siRNA-mediated knockdown of these proteins resulted in an antiproliferative effect in TCam-2, NTERA-2, and an additional embryonal carcinoma-like cell line, NCCIT. In summary, this study represents a proteomic resource for the discrimination of malignant germ cell tumor subtypes and the observed antiproliferative effect after knockdown of selected proteins paves the way for the identification of new potential drug targets.

## 1. Introduction

Germ cell tumors (GCT) are the most common malignancies in men between 15 and 40 years of age, and the incidence has constantly increased over the last four decades [1]. Germ cell tumors are histologically and clinically divided into seminomas and nonseminomas. Nonseminomas can be further subdivided into embryonal carcinomas, yolk sac tumors, chorionic carcinomas, and teratomas [2]. Seminomas and nonseminomas have a common precursor called germ cell neoplasia in situ (GCNIS) [3]. The International Germ Cell Cancer Collaborative Group (IGCCCG) developed a prognostic classification system, which divided patients with germ cell tumors into good-, intermediate, and poor-risk groups. It is based besides on several points such as the primary site of the GCT, metastatic sites of involvement, and levels of serum tumor markers in particular upon the histology of the tumors (seminoma versus non-seminoma). Because the treatment of these tumors is different, it is important to differentiate between seminomas and nonseminomas [4]. Patients even with metastasized disease can be cured in about 80% of cases by cisplatin-based chemotherapy [5, 6].

Several cell lines are available as models for the different types of GCT. NTERA-2 and NCCIT display embryonal carcinoma characteristics; meanwhile, TCam-2 is considered a model for seminoma [7, 8]. In this study, we set out to establish new biomarkers for the differentiation of GCT cell lines and formalin-fixed and paraffin-embedded (FFPE) tissue samples and to identify new potential drug targets to improve the therapeutic options especially of patients with embryonal carcinoma.

van der Zwan et al. performed a comprehensive study to identify epigenetic footprints in TCam-2 and NCCIT cell lines. They investigated interactions between gene expression, DNA CpG methylation, and posttranslational histone modifications to elucidate their role in the pathophysiology and etiology of germ cell tumors [9]. However, as the correlation between genetic alterations, RNA expression, and protein expression is highly influenced by transcriptional, translational, and posttranscriptional regulations [10], we aimed for a global, unbiased, and quantitative analysis of the two cell lines TCam-2 and NTERA-2 on the protein level.

With markers such as SALL4, OCT3/4, SOX-2, or SOX-17, numerous good and reliable diagnostic markers are available to differentiate between the different GCT subtypes [2, 11]. Regardless of this, it is of great importance to detect differences in tumor biology in order to gain a better understanding of the pathological processes of germ cell tumors. We reasoned that a proteomic approach, rather than genomic and transcriptomic studies, can identify biological differences and may also provide new potential targets for a molecular targeted therapy. For this purpose, we employed high-resolution mass spectrometric analysis combined with stable isotope labelling with amino acids in cell culture (SILAC) to visualize them in human testis and human germ cell tumor tissue [12]. This strategy can help to minimize variation occurring as a result of sample handling, because the labelling occurs in a very early stage of the experiment [13].

## 2. Material and Methods

### 2.1. Culture of TGCT Cell Lines

In the present study, the human GCT cell lines NTERA-2 (representing an embryonal carcinoma, CRL 1973; from American Type Culture Collection, Manassas, VA, USA), NCCIT (representing an embryonal carcinoma, CRL 2073; from American Type Culture Collection, Manassas, VA, USA), and TCam-2 (representing a seminoma; generously provided by the Department of Developmental Pathology, University of Bonn Medical School, Germany) were cultured in HEPES-buffered RPMI-1640 (Biochrom, Berlin, Germany) supplemented with fetal calf serum (FCS, 10%; CC Pro, Neustadt, Germany), penicillin (100 IU/ml; Sigma-Aldrich, Munich, Germany), streptomycin (100 *μ*g/ml; Sigma-Aldrich), and L-glutamine (2 mM; Biochrom, Berlin, Germany). The incubation temperature was 37°C in a humid atmosphere with 5% carbon dioxide in the air.

### 2.2. Proteomic Analysis

Stable isotope labeling with amino acids in cell culture (SILAC) and quantitative mass spectrometry were performed as described before [14–16]. TCam-2 and N-Tera2 cells were cultured in RPMI 1640 medium lacking arginine and lysine (Pierce) supplemented with 10% dialyzed FCS (Invitrogen), 4 mM glutamine, and antibiotics. “Heavy” and “light” media were distinguished by adding 0.115 mM ^13^C_6_^14^N4 L-arginine and 0.275 mM L-lysine-4,4,5,5-D4 (Eurisotop) or equimolar levels of the corresponding nonlabeled (light) amino acids (Merck Millipore), respectively. For cell lysis, 0.5% Nonidet P-40 buffer containing 50 mM Tris/HCl, pH 7.8, 150 mM NaCl, 1 mM Na_3_VO_4_, 1 mM NaF, 0.2% lauryl maltoside, and protease inhibitors (Complete, Roche) was used. Protein concentration was determined with DC Protein Assay (Bio-Rad) following the manufacturer's instructions. Equal amounts of protein of light-labeled TCam-2 were mixed with heavy-labeled NTERA-2 and vice versa. Proteins were separated by 1D-PAGE (4 to 12% NuPAGE Bis-Tris Gel, Invitrogen). After Coomassie brilliant blue staining, the gel was divided in 23 slices. Encompassing proteins were reduced with 10 mM DTT for 55 min at 56°C, alkylated with 55 mM IAA for 20 min at 26°C, and gel-digested with modified trypsin (Promega) overnight at 37°C.

Resulting peptides were separated by a C18 precolumn (2.5 cm, 360 *μ*m o.d., 100 *μ*m i.d., Reprosil-Pur 120 Å, 5 *μ*m, C18-AQ, Dr. Maisch GmbH) at a flow rate of 10 *μ*l/min and a C18 capillary column (20 cm, 360 *μ*m o.d., 75 *μ*m i.d., Reprosil-Pur 120 Å, 3 *μ*m, C18-AQ, Dr. Maisch GmbH) at a flow rate of 300 nl/min, with a gradient of acetonitrile ranging from 5 to 35% in 0.1% formic acid for 90 min using an Proxeon nano LC coupled to an Q Exactive mass spectrometer (Thermo Electron). MS conditions were as follows: spray voltage, 1.8 kV; heated capillary temperature, 270°C; and normalized collision-energy (NCE), 28. An underfill ratio of 1.2% and intensity threshold of 4.0 e4 were used. The mass spectrometer automatically switched between MS and MS/MS acquisitions (data-dependent mode). Survey MS spectra were acquired in the Orbitrap (*m*/*z* 350–1600) with the resolution set to 70 000 at *m*/*z* 200 and automatic gain control target at 2 × *e*^5^. The 15 most intense ions were sequentially isolated for HCD MS/MS fragmentation and detection. Raw data were analyzed with MaxQuant (version 1.3.0.5) using Uniprot human (version 27.08.2012 with 86725 entries) as a sequence database. Up to two missed cleavages of trypsin were allowed. Oxidized methionine was searched as variable modification and cysteine carbamidomethylation as fixed modification. The modifications corresponding to arginine and lysine labeled with heavy stable isotopes were handled as fixed modifications. The false positive rate was set to 1% at the peptide level, the false discovery rate was set to 1% at the protein level, and the minimum required peptide length was set to six amino acids.

### 2.3. Selection of Proteins for Further Investigations

To see if the proteins detected in the SILAC assays are also expressed in human testis tumor tissue, we compared the significantly expressed proteins of our results with the data in the online database *The Human Protein Atlas* (https://www.proteinatlas.org/) [17]. In the selection, we exemplarily opted for proteins that were expressed in tumor-free testicular tissue, with the consideration that these proteins could possibly be of particular importance in testicular tumors.

### 2.4. Western Blot Analysis

Total protein lysates were prepared using RIPA buffer with protease inhibitors (Roche, Germany) and were quantified by the Bio-Rad DC Protein Assay (Bio-Rad, USA). For western blotting, the following primary antibody dilutions were used: monoclonal mouse anti-CD81 (Tetraspanin-28) (Santa Cruz, sc-166029; 1 : 250), monoclonal mouse anti-PHF6 (PHD finger protein 6) (Santa-Cruz, sc-365237; 1 : 500), polyclonal rabbit anti-CBX-3 (chromobox protein homolog 3) (HPA 004902, Sigma-Aldrich; 1/250), and polyclonal rabbit anti-ENSA (alpha-endosulfine) (HPA 051292, Sigma-Aldrich: 1/500). Primary antibodies were detected by polyclonal immunoglobulins/HRP secondary antibodies (1 : 1000, Dako, DK). Membranes were developed using the ECL system (Amersham Bioscience, Germany).

### 2.5. Gene Ontology and Network Analysis

Gene ontology classification has been performed using either the Metacore software (https://portal.genego.com/) or the R package clusterProfiler. Differentially expressed proteins were loaded into Metacore software, and significantly enriched biological processes, molecular functions, and pathway networks were extracted.

Network analyses were performed using the Metacore (https://portal.genego.com/) software for the enrichment of the shortest pathways inside the group of differentially regulated proteins. The shortest path algorithm connects the differentially expressed proteins identified in the proteomic approach with additional information from the Metacore database along a directed path and potentially involved pathways. Next, most significant networks were loaded into Cytoscape software (http://www.cytoscape.org/) for further visualization. Networks were visualized using hierarchical layout in Cytoscape.

### 2.6. Tissue Samples of Primary TGCT

Formalin-fixed and paraffin-embedded tumor tissues of orchiectomy specimens were collected from 59 male patients from the University Medical Centre Göttingen, Germany. Tumors were classified and staged on the basis of the WHO classification [18]. In the present study, a number of 75 blocks have been included. Investigated cases included normal testis adjacent to tumor (*n* = 16), GCNIS (*n* = 18), seminomas (*n* = 21), and embryonal carcinomas (*n* = 20). Ethical approval for using the human material in the present study was obtained from the Ethics Committee of the University Medical Centre Göttingen.

### 2.7. Immunohistochemistry

Immunohistochemical reactions were performed on 4 *μ*m formalin-fixed and paraffin-embedded testis tissue sections. In heat-induced epitope retrieval, the antigen retrieval was carried out at 98°C in citrate buffer (low pH 6; 40 minutes) or EDTA buffer (high pH 9; 20 minutes). The primary antibodies were incubated for 30 minutes at room temperature. The following antibodies and dilutions were applied: anti-CD81 (mouse, high buffer, 1 : 200, Santa Cruz, sc-166029), anti-PHF6 (mouse, high buffer, 1 : 200, Santa-Cruz, sc-365237), anti-CBX-3 (rabbit, diluted 1/200, high buffer, HPA 004902, Sigma-Aldrich), and anti-ENSA (rabbit, diluted 1/50, low buffer, HPA 051292, Sigma-Aldrich). Afterwards, the sections were incubated with a ready-to-use HRP-labeled secondary antibody at room temperature for 25 minutes (anti-rabbit/mouse, produced in goat; Dako REAL EnVision Detection System, DAKO). The substrate DAB+ Chromogen system produces a brown end product and is applied to visualize the site of the target antigen (Dako REAL DAB+ Chromogen, DAKO). Tissue samples were counterstained with Meyer's hematoxylin (Dako) for 8 minutes and were analyzed by light microscopy.

Two independent investigators evaluated all tissue sections stained for CD81, CBX-3, PHF-6, and ENSA using an immunoreactivity staining score (IRS) as described previously [18, 19]. The percentage of positively stained cells was first classified using a 0–4 scoring system: score 0 = 0% positive cells, score 1 = less than 10% positive cells, score 2 = 10–50% positive cells, score 3 = 51–80% positive cells, and score 4 = >80% positive cells. The intensity of staining was evaluated on a four-tiered scale (0 = negative, 1 = weak, 2 = intermediate, and 3 = strong). Afterwards, the scores of intensity and staining were multiplied, and the mean value per patient was calculated, as described previously [18]. Differences of IRS between the different subtypes of GCT were statistically evaluated using Student's *t*-test (GraphPad Software, San Diego, CA, USA). A *p* value of <0.05 was considered significant.

### 2.8. siRNA Transfection

Tumor cells were transfected with 100 *μ*l transfection mix (12 *μ*l HiPerFect, Qiagen, Hilden, Germany, 2.5 *μ*l siRNA (20 *μ*M), and 85.5 *μ*l RPMI medium). siRNAs used were Hs_PHF6_10, SI05120745 and Hs_PHF6_11, SI05120752; Hs_CD81_6, SI02777236 and Hs_CD81_7, SI02777243; Hs_CBX-3_6, SI02665222 and Hs_CBX-3_7, SI03028165; and Hs_ENSA_20, SI05062218 and Hs_ENSA_21, SI05062225 (Qiagen, Hilden, Germany). After incubation for 20 min, 100 *μ*l siRNA medium were mixed with 2.3 ml culture medium to create a concentration of 1 : 1000. The cells were incubated for 24 h or 48 h.

### 2.9. Measurement of Cell Proliferation

1 × 10^5^ to 3 × 10^5^ cells were plated as described above. After 48 h incubation time, the culture medium was exchanged for a siRNA medium (2.3 ml culture medium, 100 *μ*l siRNA-Mix). For cell viability analysis, equal numbers of cells were seeded into 96-well flat-bottom plates and incubated for indicated time points. Cell viability was determined by the CellTiter 96 AQueous One Solution Cell Proliferation Assay (MTS) (Promega) according to the manufacturer's instructions.

## 3. Results

### 3.1. Quantitative Proteomic Profiling of TCam-2 and NTERA-2

**Figure 1:**
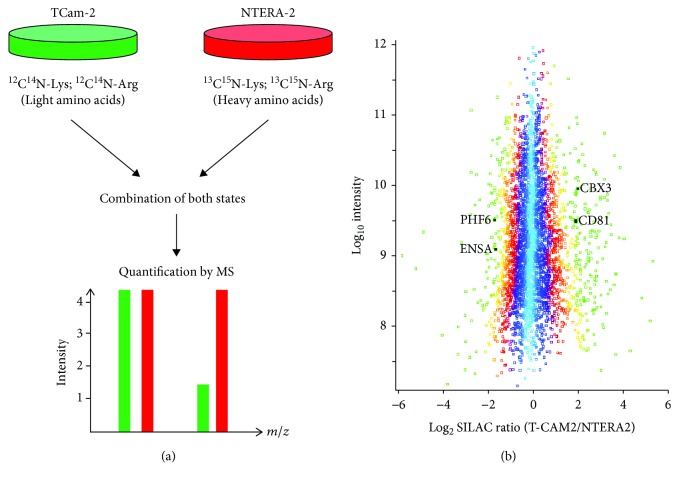
Proteomic profiling of testicular germ cell cancer cell lines. Workflow of SILAC-based mass spectrometry experiments. TCam-2 and NTERA-2 were metabolically labeled with amino acids of different masses allowing a comprehensive relative quantification of protein expression by mass spectrometry (a). Distribution of SILAC ratios of all quantified proteins according to their relative expression in NTERA-2 and TCam-2 (b).

In order to find differences in the global proteome of seminoma-like and embryonal carcinoma-like cell lines, we performed a quantitative protein expression analysis by SILAC-based mass spectrometry. The cell lines TCam-2 (representing cell line with seminoma characteristics) and NTERA-2 (representing a cell line with embryonal carcinoma characteristics) were cultured with light and heavy isotope-labeled amino acids as described in the Material and Methods. After cell lysis, equal amounts of protein of light-labeled TCam-2 were mixed with heavy-labeled NTERA-2 and vice versa and subsequently analyzed by high-resolution mass spectrometry. Due to the incorporation of SILAC amino acids, proteins derived from both cell lines can be accurately assigned to the two cell lines and its expression and can be comparatively quantified (Figure 1(a)). In two biological replicates, a total of 4856 proteins were identified and 3936 proteins could be quantified with a Gaussian distribution of the ratios between the two cell lines (Figure 1(b)). Next, an outlier significance score depending on intensity values (significance *B* in Perseus, see citation for more details [20, 21]) was calculated for every protein. 347 proteins showed a significantly different expression and 196 (TCam-2) and 102 (NTERA-2) proteins showed an at least 2.5-fold increased expression. The complete list of identified proteins detected by the quantitative MS is given in supplementary [Supplementary-material supplementary-material-1], and the complete list of differentially expressed proteins is given in supplementary [Supplementary-material supplementary-material-1].

### 3.2. Bioinformatics Analysis Reveals Differential Pathway Activation Patterns

The proteins showing significantly differential expression based on the results of the quantitative proteomic approach were subjected to a GO term analysis by the Metacore software (https://portal.genego.com/). The regulated proteins were found to be involved in different biological processes such as the regulation of tissue development or ion transport.

**Figure 2:**
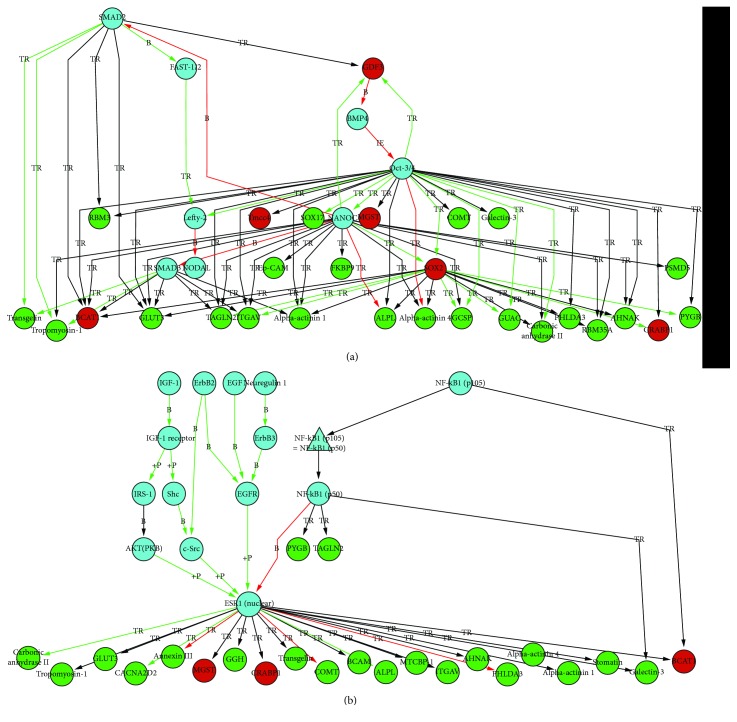
Results of network analysis: differentially regulated proteins from the SILAC approach are indicated in green (TCam-2) or in red (NTERA-2). Blue proteins represent proteins that were added from the Metacore database as potentially linked to the proteins from the SILAC analysis which are involved e.g. in gastrulation, endoderm development or formation of primary germ layer (a) and which are involved, e.g., in mammary gland development or fibroblast growth factor receptor signaling pathway (b). TR: transcription regulation; +P: phosphorylation; B: binding.

Network analysis was also performed using the Metacore (https://portal.genego.com/) software for the enrichment of the shortest pathways inside the groups of differentially regulated proteins, as described above. Supplementary [Supplementary-material supplementary-material-1] gives an overview of the networks found to be the most significantly involved. Inside the networks, differentially regulated proteins identified by the MS approach are indicated in green for TCam-2 (representing seminomatous histology) or in red for NTERA-2 (representing nonseminomatous histology). Blue proteins represent proteins that were added from the Metacore database as potentially linked to the proteins derived from the MS analysis which are involved in different processes such as proteins which play a crucial role, for example, in gastrulation, endoderm development, or formation of a primary germ layer (Figure 2(a)) or mammary gland development or fibroblast growth factor receptor signaling pathway (Figure 2(b)).

### 3.3. In Vitro and In Vivo Validation of Differentially Expressed Proteins

To validate the results of the proteomics approach, four proteins were selected showing significant differences in expression between the two GCT-cell lines and which showed a physiological expression in nonneoplastic germ cells or several stages of spermatogenesis (according to The Human Protein Atlas database [17]), namely, CD81, CBX-3, PHF-6, and ENSA. In order to verify the differential protein expression, we additionally examined a third cell line named NCCIT (showing EC characteristics also).

**Figure 3:**
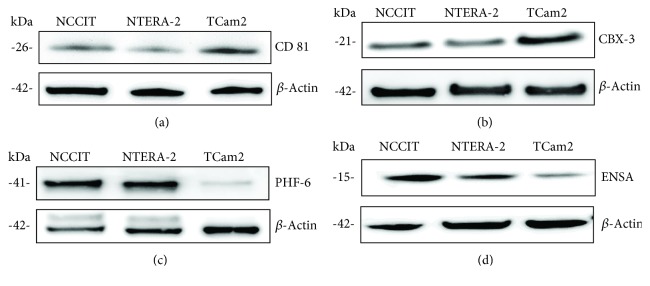
In vitro validation of differentially expressed proteins. In line with the results of the SILAC method and mass spectrometry, CD81 and CBX-3 show markedly higher expression in TCam-2 than in NTERA-2 (a, b). Conversely, PHF-6 and ENSA show markedly higher expression in NTERA-2 than in TCam-2 cells (c, d).

Western blot analyses confirmed the results of the quantitative proteomic profiling. According to MS, the amount of CD81 (ratio *TCam* − 2/NTERA − 2 = 3.625) and CBX-3 (ratio *TCam* − 2/NTERA − 2 = 3.281) protein was significantly higher in TCam-2 than in NTERA-2- cells (supplementary [Supplementary-material supplementary-material-1]). These differences could be reproduced by western blot analysis for CD81 and CBX-3 which showed a marked difference in expression between the two cell lines with embryonal carcinoma characteristics (NTERA-2- and NCCIT) and TCam-2 (seminoma characteristics) (Figures 3(a) and 3(b)). In contrast, expression of PHF6 (ratio NTERA − 2/*TCam* − 2 = 3.841) and ENSA (ratio NTERA − 2/*TCam* − 2 = 2.707) proteins was significantly higher in NTERA-2 than in TCam-2 cells (supplementary [Supplementary-material supplementary-material-1]). These differences could also be reproduced by western blot analysis for PHF6 and ENSA, which showed marked differences in protein expression in NTERA-2 and NCCIT compared to TCam-2 (Figures 3(c)and 3(d)). The results confirmed the same expression pattern of NCCIT- and NTERA-2 cells in contrast to TCam-2 cells.

To confirm the results of the western blot analyses and gain insight into expression patterns within single cells of primary tumors (i.e., nuclear, cytoplasmic, or membranous staining), commercially available antibodies against CD81, CBX-3, PHF-6, and ENSA were used for immunohistochemical analysis of human tissues. FFPE tissue samples of 59 patients with malignant GCT of the testis were investigated by immunohistochemical analysis. The investigated samples comprised tumor-free testicular tissue (*n* = 16), GCNIS (*n* = 18), seminomas (*n* = 21), and embryonal carcinomas (*n* = 20). Seminomas (to compare with TCam-2 cells) and embryonal carcinomas (to be compared with NTERA-2 and NCCIT cells) were explicitly chosen for direct a comparison with the cell lines.

**Figure 4:**
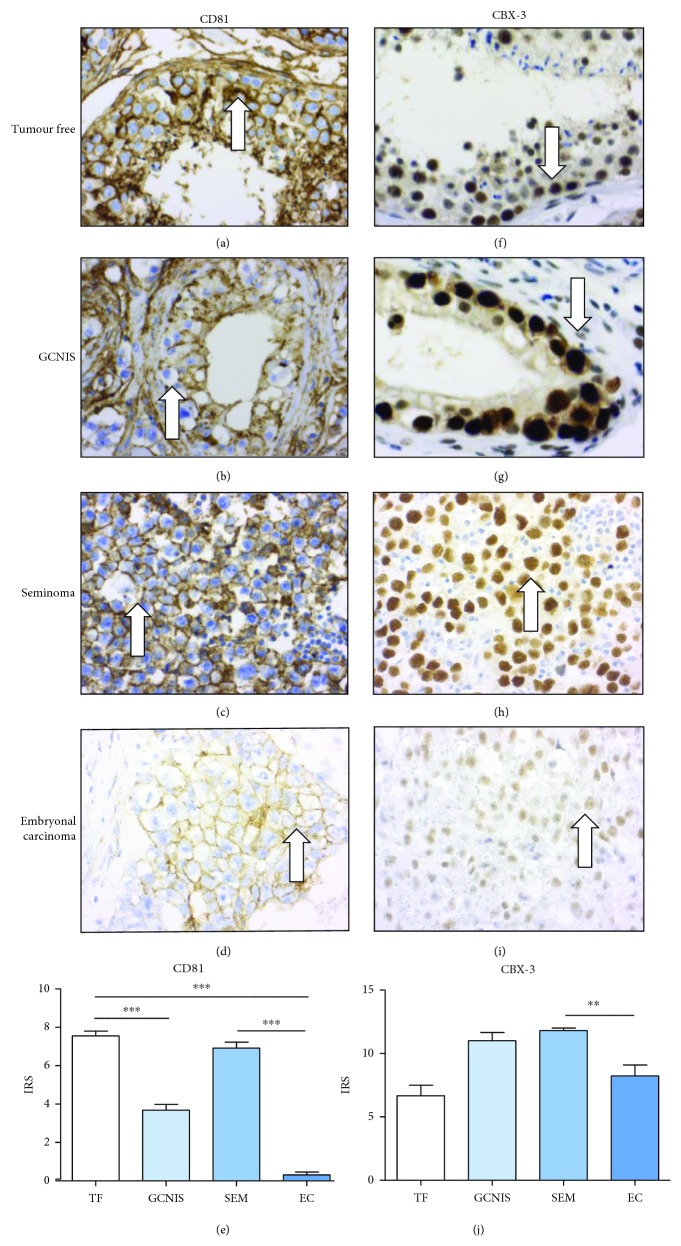
Immunohistochemical analysis of selected proteins (more highly expressed in TCam-2 than in NTERA-2) in tumor-free testis, GCNIS, seminoma, and embryonal carcinoma: a tumor-free testis and GCNIS show no differences in CD81 and CBX-3 >expression (a, b, f, g). The expression of CD81 and CBX-3 in embryonal carcinomas (d, i) is significantly lower than that in seminomas (c, h). The differences between seminomas and embryonal carcinomas in staining intensity (IRS) of CD81 (i) and CBX-3 (j) are significant.

A tumor-free testis showed a strong membranous and cytoplasmic expression of CD81 protein (Figure 4(a), arrow). In germ cell neoplasia *in situ* (GCNIS), a similar pattern of membranous and cytoplasmic expression was found (Figure 4(b), arrow). All investigated seminomas showed a strong membranous expression of CD81 in tumor cells (Figure 4(c), arrow). In contrast, no or only a weak staining of CD81 was seen in embryonal carcinoma cells (Figure 4(d), arrow). The differences in staining intensity (according to the immunoreactivity staining score (IRS)) of CD81 (Figure 4(e)) observed between seminomas and embryonal carcinomas were marked and showed a statistical significance.

CBX-3 protein showed a strong nuclear expression in spermatogonia, in contrast to a weaker expression in spermatocytes and spermatids (Figure 4(f), arrow). In addition, a strong nuclear expression of CBX-3 was observed in GCNIS (Figure 4(g), arrow). A strong nuclear expression was also detected in most seminoma cells (Figure 4(h), arrow). A much weaker nuclear expression of CBX-3 was seen in embryonal carcinomas (Figure 4(h), arrow). The differences in staining intensity (according to the immunoreactivity staining score (IRS)) of CBX-3 (Figure 4(j)) observed between seminomas and embryonal carcinomas were marked and showed a statistical significance. The results of CD81 and CBX-3 immunohistochemistry confirmed the findings of the proteomic and western blot analyses.

**Figure 5:**
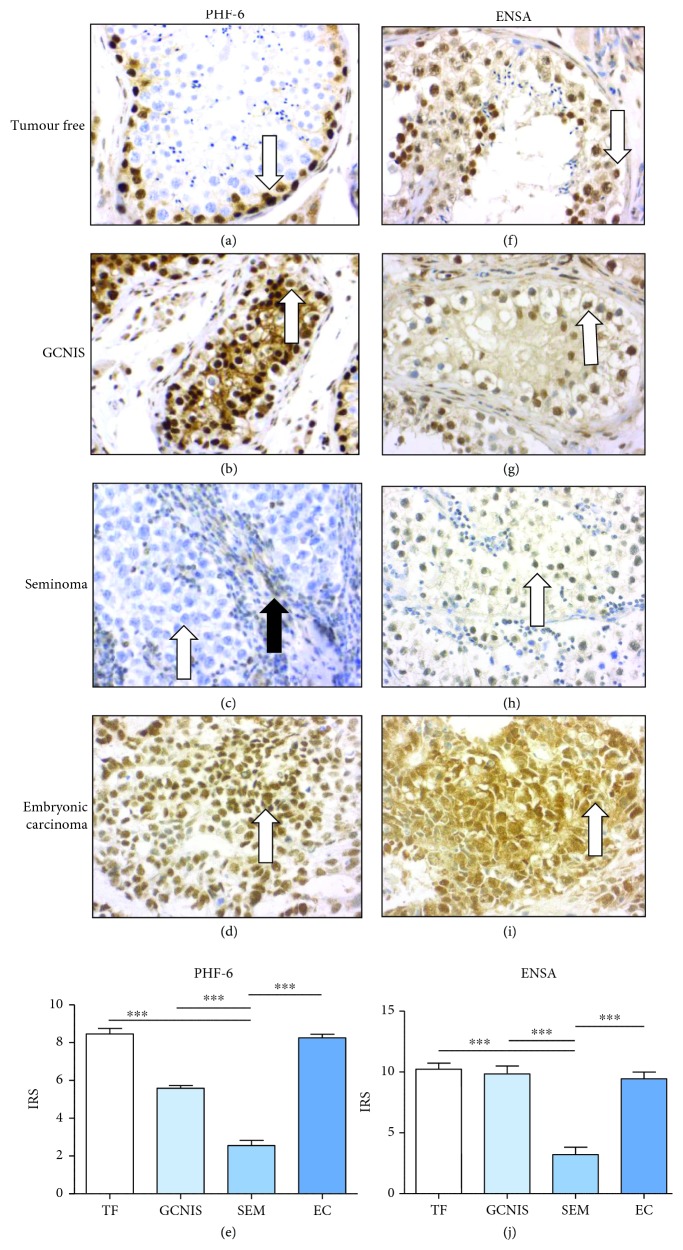
Immunohistochemical expression of selected proteins (more highly expressed in NTERA-2 than in TCam-2) in a tumor-free testis, GCNIS, seminoma, and embryonal carcinoma: A tumor-free testis and GCNIS show no marked differences in protein expression of PHF-6 and ENSA on immunohistochemical analysis (a, b, f, g). The expression of PHF-6 and ENSA in embryonal carcinomas (d, i) is markedly higher than that in seminomas (c, h; white arrow tumor cells, black arrow lymphocytes). The differences between seminomas and embryonal carcinomas in staining intensity (IRS) of PHF-6 € and ENSA (j) are significant.

Immunohistochemically, PHF-6 protein showed strong nuclear expression in spermatogonia and slightly weaker expression in sertoli cells, whereas later stages of spermatogenesis did not express PHF-6 (Figure 5(a), arrow). The expression of PHF-6 in GCNIS was heterogeneous, with some nuclei showing strong and others weak or no expression (Figure 5(b), arrow). Seminomas showed no or only minimal expression of PHF-6 protein. Beside the tumor cells, lymphocytes also expressed PHF-6 (Figure 5(c), arrows). In contrast, embryonal carcinoma cells showed a strong nuclear expression of PHF-6 (Figure 5(d), arrow). The differences in staining intensity (according to the immunoreactivity staining score (IRS)) of PHF-6 (Figure 5(e)) observed between embryonal carcinomas and seminomas were marked and showed a statistical significance.

ENSA showed a strong nuclear and weak cytoplasmic expression in late stages of spermatogenesis, whereas spermatogonia were only weakly positive for ENSA (Figure 5(f), arrow). In addition, ENSA was found to be strongly expressed in GCNIS, mostly nuclear (Figure 5(g), arrow). Seminoma cells showed only a moderate nuclear expression of ENSA (Figure 5(h), arrow). Interestingly, nuclear as well as cytoplasmic expression of ENSA in embryonal carcinomas was significantly stronger than that in seminomas (Figure 5(i), arrow).

The differences in staining intensity (according to the immunoreactivity staining score (IRS)) of ENSA (Figure 5(j)) observed between embryonal carcinomas and seminomas were marked and showed a statistical significance. The results of PHF-6- and ENSA-immunohistochemistry confirmed the proteomic and western blot findings.

### 3.4. Knockdown of Selected Proteins Results in Reduction of Cellular Survival in Seminoma and EC Cell Lines

**Figure 6:**
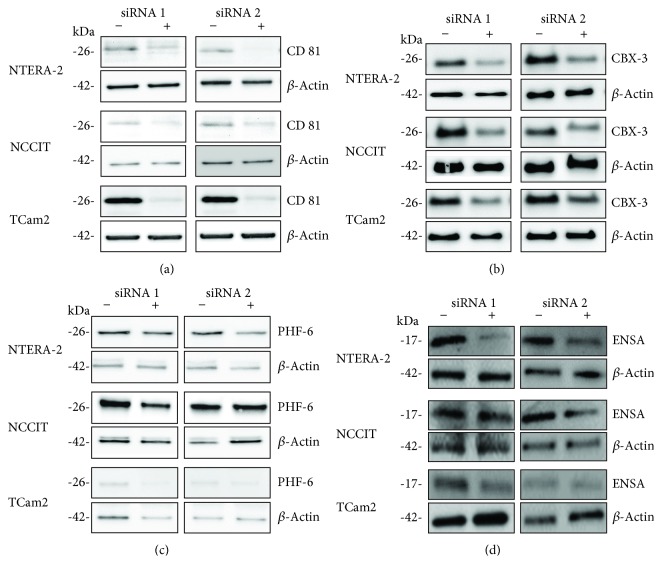
Transfection with siRNA markedly reduces protein expression of CD81, CBX-3, PHF-6, and ENSA: NCCIT, NTERA-2, and TCam-2 were transfected with a siRNAs against CD81, CBX-3, PHF-6, and ENSA. The protein expression was markedly reduced after transfection with siRNA (a–d).

To gain insight into involved cellular processes and the putative role of selected proteins detected by MS, siRNA experiments were performed. The tumor cell lines NCCIT, NTERA-2, and TCam-2 were transfected with two specific siRNAs to achieve knockdown of CD81, CBX-3, PHF-6, and ENSA as described above. A marked downregulation of protein expression was achieved for all proteins for both siRNAs used (Figures 6(a)–6(d)).

**Figure 7:**
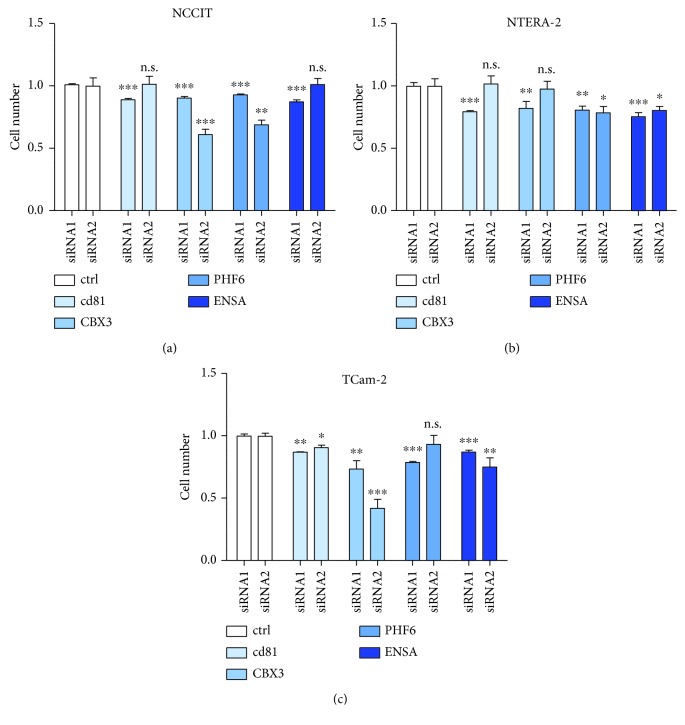
Transfection with siRNA decreases cell proliferation of GCT cell lines: in all investigated tumor cell lines, NCCIT (a), NTERA-2 (b), and TCam-2 (c), proliferation was significantly reduced after transfection with siRNA against CD81, CBX-3, PHF-6, and ENSA.

This downregulation had no effect on proliferation after 24 h (data not shown). 48 h after downregulation of CD81, we observed a significant decrease of proliferation in NTERA-2, NCCIT, and TCam-2 cells. In NCCIT and NTERA-2, transfection with siRNA2 showed no statistical significances in proliferation (Figures 7(a)–7(c)). After the downregulation of CBX-3, we also observed a significant decrease of proliferation in all investigated GCT cell lines. SiRNA2 showed no statistical significances in proliferation in NTERA-2 cells (Figures 7(a)–7(c)). After the downregulation of PHF-6, we observed a significant decrease of proliferation in all investigated GCT cell lines, too. SiRNA2 had no effect in proliferation in TCam-2 cells (Figures 7(a)–7(c)). Finally, we observed a significant decrease of proliferation in all investigated GCT cell lines after downregulation of ENSA. SiRNA2 showed no statistical significances in proliferation in NCCIT cells (Figures 7(a)–7(c)). The decrease in proliferation after siRNA transfection appears to be independent of the different levels of expression of the proteins, as one might suspect.

## 4. Discussion

GCTs are highly interesting tumors, both from a point of view of developmental biology and considering their tumor biology. GCTs are divided into seminomas and nonseminomas [2]. For *in vitro* studies, several GCT cell lines are well established, such as TCam-2 (with seminoma characteristics), NTERA-2, and NCCIT (both with characteristics of embryonal carcinomas) [7, 8, 22, 23]. We aimed to identify new biomarkers for the differentiation of GCT cell lines on the protein level. A recent study by van der Zwan et al. identified epigenetic footprints in TCam-2 and NCCIT cell lines. These analyses confirmed a more germ cell-like profile in TCam-2 cells and, in contrast, a more pluripotent phenotype in NCCIT cells [9]. We compared the results of a differential gene expression in TCam-2 and NCCIT cells of the work by van der Zwan et al. [9] to the results of our project and found 44 (TCam-2) and 23 (NTERA-2 or NCCIT) similarly significantly differentially expressed proteins/genes. The exact names of the genes are listed in Table 1. This underpins the potential importance of these genes in the biology of these tumor cells.

In addition, we searched for proteins that were differently expressed in cell lines and that are expressed physiologically in spermatogonia and later stages of spermatogenesis. Furthermore, we analyzed if the findings from the cell line experiments were reproducible in FFPE tissue samples of human GCTs. In the present study, 111 proteins showed a statistically significant two-fold increase in expression in TCam-2 compared to NTERA-2. Influenced by the expression pattern in spermatogenesis and germ cell tumors (according to The Human Protein Atlas database [17]) by the availability of antibodies and their applicability on both, cell lines and FFPE tissues, we chose the proteins CD81 (ratio from SILAC-analysis TCam-2/NTERA-2: 3.626) and CBX-3 (ratio TCam-2/NTERA-2: 3.282) for further investigations.

CD81 is a cell surface protein of the tetraspanin family. It is widely expressed on many healthy tissues and on the majority of tumor cells. Vences-Catalan et al. demonstrated in comprehensive studies the role of CD81 as a promoter of tumor growth and metastasis with a putatively important role in tumor progression [24, 25]. Zhang et al. described that an increased expression of CD81 was significantly associated with reduced overall survival in patients with mammary carcinoma. Furthermore, CD81 knockdown results in decreased proliferation and migration in mammary carcinoma cell lines in vitro [26]. In addition, Hong et al. described that CD81 increases melanoma cell motility by upregulating the metalloproteinase MT1-MMP-expression. This could be explained by a prooncogenic Akt-dependent Sp1 activation [27]. Interestingly, in our study, we could demonstrate that CD81 showed marked differences in its expression when comparing seminoma (high expression) and embryonal carcinoma tissue samples (low expression). CD81 interacts with CD9 (another member of the tetraspanin family), which is also significantly upregulated in TCam-2 cells (supplementary [Supplementary-material supplementary-material-1]), and both play an important role in the TGF beta signaling pathway in melanoma cells [28]. TGF beta, EGF, and FGF have been shown to play a role in the differentiation of TCam-2 into a cell type resembling a mixed nonseminoma [7]. This would be in line with the findings of our study, as shown in Figure 2, where involved networks are displayed and demonstrate that these growth factor signaling pathways play a crucial role. In addition, first investigations with two siRNAs against CD81 showed a significant reduction of proliferation in TCam-2 cells, as well as in NTERA-2 and NCCIT cells (Figure 7). So the extent of proliferation seems not to correlate with the expression level of CD81. Usually, TCam-2 cells proliferate remarkably slower than NTERA-2 and NCCIT. However, the high expression of CD81 on TCam-2 and seminoma samples is interesting given the induction of nonseminoma-like phenotype by TGF beta signaling [6]. However, the exact mechanism on how the proliferation was reduced in this cell line has not been investigated in more detail and remains to be elucidated.

A second protein which was markedly higher expressed in TCam-2 cells than in NTERA-2 and NCCIT cells is CBX-3. Little is known about the function of CBX-3 in cancer cells. One study could demonstrate the essential function of CBX-3 for male germ cell survival and spermatogenesis [29]. Ma et al. described very recently that the expression of CBX-3 in osteosarcomas is associated with a large tumor size, high distant metastasis rate, and high clinical stage rate. Furthermore, they could show that knockdown of CBX-3 by siRNA results in increased apoptosis and cell cycle arrest at the G0 and G1 phases [30]. Another recent study demonstrates the role of CBX-3 in tumor progression in pancreatic cancer cell lines. It could be shown that the tumor-promoting effect of CBX-3 might be mediated by CDK1 [31]. Further similar results were demonstrated by Zhang et al. which demonstrate that a high expression of CBX-3 in squamous carcinomas of the tongue is associated with poor prognosis. In addition, the inhibition of CBX-3 leads to cell cycle delay via the p21 pathway [32]. Similar findings were described by Fan et al. who showed that CBX-3 promotes the progression of the cell cycle and proliferation *in vitro* and *in vivo* in colon cancer cells. They could explain that CBX-3 promotes colon cancer cell proliferation by curbing cell cycle G1-S phase transition [33]. Another study showed a high expression of CBX-3 in various human cancer tissues and suppression of tumor growth of various cancer-derived cell lines following siRNA-mediated knockdown [34]. Again, we found that two siCBX-3 reduced tumor cell growth in all investigated GCT cell lines. Further investigations of the mechanisms underlying this observation and a potential role in antitumor therapy are pending.

Our investigations furthermore showed markedly higher expression of PHF-6 in NTERA-2 and NCCIT cell lines as compared to TCam-2. PHF-6 is a gene found in association with the *Börjeson-Forssman-Lehmann* syndrome [35]. Interestingly, patients with this syndrome generally show hypogonadism [36]. Our results show a strong nuclear positivity of the PHF-6 protein in normal spermatogenesis and GCNIS, whereas the expression in seminomas was markedly lower. Notably, PHF-6 expression was markedly higher in embryonal carcinomas, than in seminomas in FFPE samples. Recently, PHF-6 has been described to be involved in regulating rRNA synthesis, which may contribute to its role in cell cycle control, maintenance of genomic integrity, and tumor suppression [37]. This would be in line with our results which show that siPHF-6 suppresses cell proliferation in GCT cell lines (Figure 7).

Finally, ENSA (alpha-endosulfine) is a potent inhibitor of PP2A-B55*δ* [38, 39]. PP2A is expressed in both primary GCTs and GCT cell lines. Its inhibition mediates an apoptosis induction in GCT cells through activation of the MEK-ERK signaling pathway [40]. Furthermore, inhibition of ENSA with two specific siRNAs leads to reduced cell proliferation in all investigated GCT cell lines. Seminoma cells showed only a moderate nuclear expression (Figure 5(h)), in contrast to embryonal carcinomas which show a significantly stronger expression of ENSA than seminomas. However, this finding supports the close developmental relationship between early spermatogenesis and seminomas in contrast to embryonal carcinomas.

Using network analysis, the differently expressed proteins identified by our proteomic analysis could be linked to the proteins assigned from the Metacore database. Proteins were closely linked to proteins such as SOX-2, SOX-17, NANOG, or OCT3/4, which all have been described to play crucial roles in pluripotency and differentiation of germ cells and germ cell tumors [41–43]. Interestingly, another putative stem cell gene, GDF-3, has been found to be expressed in both seminomas and breast carcinomas [42]. The increased expression of BCAT1 in NTERA-2 cells compared to TCam-2 cells seems also of interest in distinguishing germ cell tumor subtypes. These findings are in line with the results of Rodriguez et al., which could demonstrate a strong overexpression of BCAT-1 in nonseminomas germ cell tumors [44].

## 5. Conclusion

In summary, high-resolution mass spectrometry in combination with SILAC-quantification is suitable for the detection of differentially expressed proteins in GCT cell lines. These results could be reproduced by western blot analysis. Proteins detected as differentially expressed by SILAC-based MS could furthermore be validated in FFPE samples of human GCTs and normal (tumor-free) testes. This method is therefore valuable for the detection of new markers with the potential to distinguish between different histologic subtypes of these tumors. In addition, network analyses serve to classify the differentially expressed proteins into functional groups. Finally, siRNA results indicate an antiproliferative potential of the therapeutic knockdown of several detected proteins, which warrants their evaluation as potential therapeutic targets.

## Figures and Tables

**Table 1:** Differentially expressed genes compared with van der Zwan et al. [9] and the results of the present study. The table shows the identical genes, which are differentially expressed in NCCIT or NTERA-2 compared to TCam-2.

Increased in TCam-2	Increased in NTERA-2/NCCIT
ACSF2	ACAT2
ANXA1	AP1S2
ANXA3	ARMCX2
CACNA2D2	ARRB1
COL17A1	ASS1
COL23A1	BCAT1
COMT	C1QBP
CSRP1	CECR5
DUSP23	CRABP1
EFR3A	CTSC
ENO2	DPYSL3
EPCAM	GFPT2
FLNC	HPRT1
GLIPR2	IQGAP2
GMPR	MAD2L2
GSN	MGST1
HEG1	PFAS
HIC2	PNMA2
ITGAV	POLR3G
LAMA5	SH3BGRL
ODZ4	SOX2
PHLDA3	TMCO1
PPM1F	UGP2
PRAME	
PROM1	
PSD3	
PSTPIP2	
PVR	
PYGB	
RAB15	
RASSF2	
RCN1	
SDF2L1	
SERPINE2	
SLC25A29	
SPARC	
TAGLN	
TCL1A	
TFAP2C	
TMEM132A	
VAMP8	
VSNL1	
WASL	

## Data Availability

We have submitted our raw and processed mass-spectrometry data to the PRIDE proteomics data repository (http://www.ebi.ac.uk/pride/archive/), to allow a thorough evaluation of the results (Project accession: PXD010275; Account details: reviewer33380@ebi.ac.uk; password: TrOAL2N5).

## Supplementary Materials

Supplementary 1 Supplementary Table 1 Complete list of identified proteins detected by the quantitative mass spectrometry as revealed by MaxQuant analysis of the obtained MS raw data.

Supplementary 2 Supplementary Table 2 Differentially expressed proteins in TCam-2 and NTERA-2.

Supplementary 3 Supplementary Table 3. Significantly involved networks of proteins detected by SILAC and mass spectrometry.
